# Clinical features and outcomes of PComA aneurysms originating from fetal posterior communicating arteries in a single institution

**DOI:** 10.1186/s41016-020-00200-6

**Published:** 2020-07-01

**Authors:** Xin Chen, Hao Li, Ming-Ze Wang, Mao-gui Li, Yong Cao, Dong Zhang, Yan Zhang, Hao Wang, Shuo Wang

**Affiliations:** 1 Department of Neurosurgery, Beijing Tiantan Hospital, Capital Medical University, Beijing, 100070 China; 2 China National Clinical Research Center for Neurological Diseases, Beijing, 100070 China; 3 Beijing Key Laboratory of Translational Medicine for Cerebrovascular Diseases, Beijing, 100070 China; 4 Center of Stroke, Beijing Institute for Brain Disorders, Beijing, 100070 China

**Keywords:** Aneurysm, Fetal posterior cerebral artery, Clinical features, Outcome

## Abstract

**Background:**

The aim of this study was to retrospectively analyze our experience with the patients who underwent surgical treatment of posterior communicating artery (PComA) aneurysms originating from fetal posterior cerebral artery (fPCA) and analyze the risk factors for the postoperative radiological infarction and outcome.

**Methods:**

From 2011 to 2020, we retrospectively reviewed 74 PComA aneurysms originating from fPCA in terms of the clinical and radiological features and obtained the follow-up data from the Department of Neurosurgery, Beijing Tiantan Hospital, Capital Medical University. The relationships between these features and follow-up data were assessed with the univariate and multivariate analysis.

**Results:**

In this series, 74 aneurysms were occurring at the origin of fPCAs. All the patients showed complete obliteration of their aneurysms. Full fPCA type tends to be a predictive factor for radiological infarction (univariate *χ*^2^ = 5.873, *P* = 0.027; multivariate OR = 0.264, *P* = 0.060). Postoperative radiological infarction (univariate *χ*^2^ = 12.611, *P* = 0.001; multivariate OR = 6.033, *P* = 0.043), rupture (univariate *χ*^2^ = 4.514, *P* = 0.047; multivariate OR = 57.966, *P* = 0.044), and hypertension (univariate *χ*^2^ = 5.301, *P* = 0.024; multivariate OR = 24.462, *P* = 0.029) tend to be the independent predictive factors for poor prognosis at 3 months after discharge.

**Conclusions:**

In conclusion, we report a series of patients harboring aneurysms originating from the fPCA. Surgical clipping is a reliable strategy. Full fPCA type is related to postsurgical infarction. Postoperative radiological infarction, rupture, and hypertension tend to be the independent predictive factor for poor prognosis at 3 months after discharge.

## Background

The posterior communicating artery (PComA) plays a vital role in physiologically mediating the anterior and posterior cerebral circulation and providing a facilitating channel for reversible flow to maintain adequate cerebral perfusion. In most cases, the posterior cerebral artery (PCA) receives the blood supply predominantly from the posterior circulation [1]. Yet, a fetal variant of PCA, named fetal PCA (fPCA), whose overall incidence has been reported 3–36% among the whole population, either unilaterally or bilaterally, gets its predominant blood supply from anterior circulation [2–4]. An fPCA is called a full fPCA if the P1 segment is not visualized on computed tomography angiography (CTA), magnetic resonance angiography (MRA), or after injection of contrast into the vertebral artery; a partial fPCA if the P1 segment is smaller than the PcomA; or an intermediate fPCA if the P1 segment is as large as the PcomA.

The internal carotid artery (ICA)-PComA junction is the favorite site for intracranial aneurysms, and approximately 20 to 30% of cerebral aneurysms locate here [5–7]. ICA-PComA aneurysms, so-called PComA aneurysms, are aneurysms of the ICA occurring at the origin of the PComA [8]. Either surgical clipping or endovascular obliteration of PComA aneurysms has a big chance to inadvertently injury the PComA itself or adjacent perforating arteries, leading to ischemic injury to dependent regions [9, 10]. Especially in the cases of fetal variant circulation, the occlusion of the dominant feeder to these regions can be especially crippling, which will cause potential infarction of the midbrain, thalamus, and occipital region [5, 11, 12]. However, there were only few reports referring to surgical experiences about the fPCA aneurysms. In addition, the risk factors for the outcomes of surgical treatment for fPCA aneurysms have not been evaluated yet. Thus, the aim of this study was to retrospectively report our surgical experience with a series of 74 fPCA aneurysm patients in our institution and analyze the risk factors for the outcome.

## Methods

### Patient population and follow-up data

A total of 388 cases of PcomA aneurysms underwent microsurgery in the Department of Neurosurgery, Beijing Tiantan Hospital, Capital Medical University, from 2011 to 2020. Among these patients, 99 patients were identified with fPCA in the circle of Willis. Seventy-four out of 99 patients had their aneurysms originating from the fPCA. Clinical data were collected from the hospital records. The radiological data (including CT, CTA, and DSA) were examined on Picture Archiving and Communication Systems (PACS). Follow-up data were obtained from telephone reviews. This study was approved by the institutional review board in our hospital. All procedures performed in studies involving human participants were in accordance with the ethical standards of the institutional and/or national research committee and with the 1964 Helsinki Declaration and its later amendments or comparable ethical standards.

### fPCA identification and classification

**Fig. 1:**
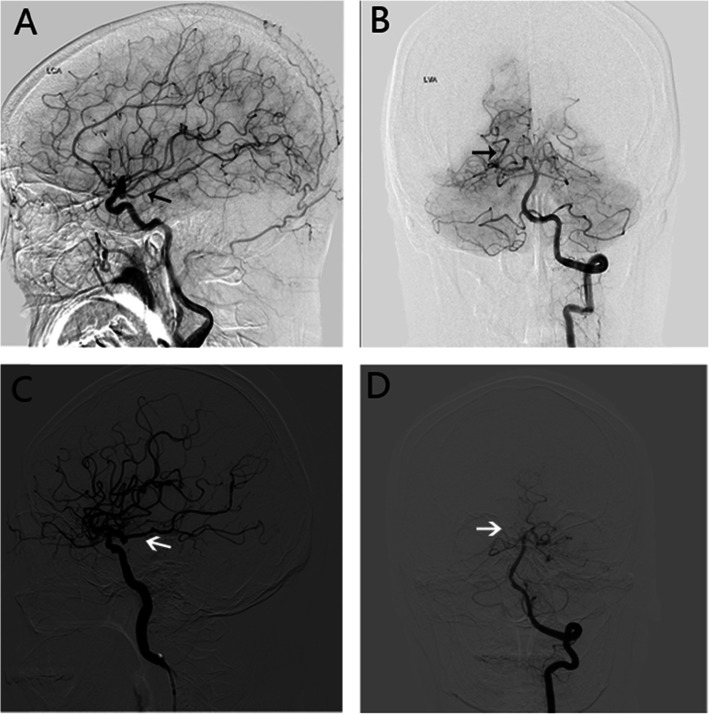
DSA demonstrating the partial fPCA and complete fPCA. **a**, **b** Representative images of partial fPCA on DSA. The arrow on **a** shows the PComA is continuous as PCA in the same diameter of the lumen, but the arrow on **b** shows the PCA is originating from the basilar artery. **c**, **d** Representative images of complete fPCA on DSA. The arrow on **c** shows PComA is continuous as PCA in the same diameter of the lumen; the arrow on **d** shows the right PComA was absent. fPCA, fetal posterior cerebral artery; PComA, posterior communicating artery; PCA, posterior cerebral artery; DSA, digital subtraction angiography

Generally speaking, the fPCA variant was defined as one with primary perfusion of the PCA vessels from ICA angiography injections and minimal perfusion of PCA vessels on vertebrobasilar angiography, often with an absent or hypoplastic P1 segment. Due to the architecture, fPCA was classified into two sorts, partial fPCA and full fPCA. For partial fPCA, P1 segment was hypoplastic and the PComA was significantly larger or the same in diameter (Fig. 1a, b), but for the regular PCA/PComA, P1 segment is significantly larger than PComA in diameter; full fPCA was defined by that the P1 segment of PCA was absent and the PComA was completely originating from ICA (Fig. 1c, d) [13]. The fPCA was determined by DSA or CTA (the patients without DSA).

### Complications and outcomes

The main complication in this study was postoperative radiological infarction. Comparing with preoperative images on CT scan, the new infarction on the brain territory supplied by PCA was defined as postoperative radiological infarction. The Glasgow Outcome Scale (GOS) score of 3 months after discharge was assessed. According to the score, the GOS was classified into good outcome (GOS score was 4 and 5) and bad outcome (GOS score was 1, 2, and 3).

### Statistical analysis

All data were analyzed by the use of SPSS statistical program (version 19.0 for Windows). The associations between clinical data and postoperative radiological infarction or outcome at 3 months after surgery were examined by Student’s *t* test for continuous variables and Pearson’s *χ*^2^ test or Fisher’s exact test for categorical variables. Then, the statistically significant parameters were enrolled into the binary logistic regression analysis to calculate the independent predictive factors. *P* < 0.05 was considered statistically significant.

## Results

### Clinical and radiological data

The clinical features of the 74 patients are summarized in Table 1. The mean age of the patients was 57.7 years (range, 24–77). There was a female preponderance with the ratio of 56/18 (female/male).

**Table 1:** Summary of clinical data of 74 patients

Demographics		No. of cases (%)
Sex	Male	18 (24.3%)
	Female	56 (75.7%)
Age in years	< 60 years	39 (52.7%)
	Age ≥ 60 years	35 (47.3%)
Hypertension	Yes	46 (62.2%)
	No	28 (37.8%)
Hunt-Hess grade	0*	26 (35.1%)
	1	25 (33.8%)
	2	14 (18.9%)
	3	9 (12.2%)
Third cranial nerve palsies	Yes	16 (21.6%)
	No	58 (78.4%)
fPCA type	Partial	29 (39.2%)
	Full	45 (60.8%)
fPCA sides	Bilateral	31 (41.9%)
	Left side	16 (21.6%)
	Right side	27 (36.5%)
Aneurysm size	Small (diameter < 0.5 cm)	15 (20.3%)
	Medium (0.5 cm ≤ diameter < 1.5 cm)	53 (71.6%)
	Large (1.5 cm ≤ diameter < 2.5 cm)	5 (6.8%)
	Giant (diameter ≥ 2.5 cm)	1 (1.4%)
Aneurysm shape	Regular	39 (52.7%)
	Irregular	35 (47.3%)
Multiple aneurysms	Yes	17 (23.0%)
	No	57 (77.0%)
Intraoperative temporary clipping	Yes	9 (12.2%)
	No	65 (87.8%)
Postoperative radiological infarction	Yes	19 (25.7%)
	No	55 (74.3%)
Postoperative complications	Yes	24 (32.4%)
	No	50 (67.6%)

*Hunt-Hess grade 0 refers to unruptured aneurysms

The mean maximal aneurysm diameter was 7.34 mm (range, 3–25 mm), and the medium type make up the vast majority (53/71.6%). Thirty-nine (52.7%) aneurysms showed regular sacciform shape, and others were irregular (lobulated or rectangular). Thirty-one (41.9%) patients showed Bilateral fPCA type, and 16 (21.6%) and 27 (36.5%) showed left and right side type, separately. The full fPCA type accounts for 45 (60.8%) of all. Seventeen patients (23.0%) had multiple aneurysms (the other aneurysms were located at ICA, middle cerebral artery, and anterior communicating artery) on preoperative angiography. Of all the patients, 9 (12.2%) patients underwent temporary clipping. Sixteen (21.6%) patients suffered from the third cranial nerve palsies, and 46 (62.2%) had the case history of hypertension. The preoperative CT scan revealed 48 (64.9%) subarachnoid hemorrhage (SAH), with Hunt-Hess grade I of 25 (33.8%), II of 14 (18.9%), and III of 9 (12.2%).

### Radiological infarction and outcomes

**Fig. 2:**
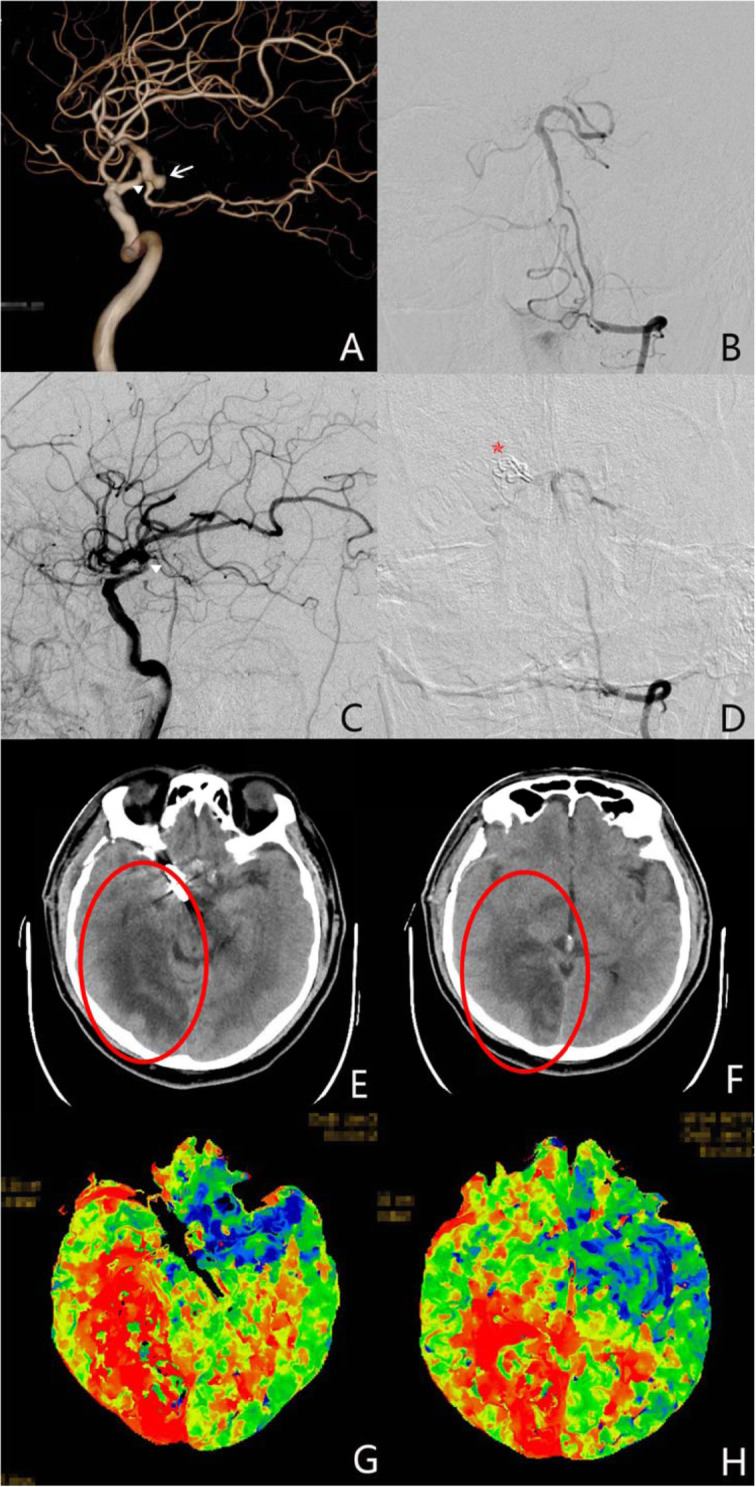
The demonstration of a male, 55-year-old case. **a** The 3D RCA development of DSA shows the PComA aneurysm originating from the origin of the full-type PCA. White arrowhead shows the aneurysm. The triangle shows the origin of full PCA type from RCA system. **b** The vertebral development of DSA shows the missing of PCA. **c** The postoperative RCA development of DSA shows no image of the PCA after surgical clipping and the aneurysm is totally eliminated. The triangle shows the preoperative origin site of full PCA type from RCA system. **d** The postoperative vertebral development of DSA shows the missing of PCA and totally occlusion of the aneurysm for **c**. Five-pointed star indicates the titanium clips. **e**, **f** The postoperative CT scan show the infarction of right temporal and occipital lobe. The red ellipses demonstrate the ischemic low density. **g**, **h** The CT perfusion imaging (axial time to peak) shows the ischemia (red area) of right temporal and occipital lobe

All the patients were identified getting complete obliterations of their aneurysms and having preservation of fPCA by postoperative DSA or CTA (Fig. 2a–d). Based on the postoperative CT scan before the patients were discharged, 19 patients (25.7%) showed radiological infarction on the PCA supplying territory (Fig. 2e–h). GOS scores for the 74 patients at the time of 3 months after discharge are shown in Table 2. Most patients (85.1%) have a GOS score of 4 and 5 defined as good outcome.

**Table 2:** The prognosis of the 74 patients at 3 months after surgery

GOS score	No. of cases (%)
5 (good)	51 (68.9%)
4 (good)	12 (16.2%)
3 (poor)	8 (10.8%)
2 (poor)	2 (2.7%)
1 (poor)	1 (1.4%)

### Predictors of postoperative radiological infarction and poor functional outcome

For postoperative radiological infarction, aneurysm size (*t* = − 2.482, *P* = 0.015), fPCA type (*χ*^2^ = 5.873, *P* = 0.027), and intraoperative temporary clipping (*χ*^2^ = 4.794, *P* = 0.043) showed dependencies.

For the outcome of 3 months after discharge, aneurysm size (*t* = − 2.492, *P* = 0.016), age (*t* = − 2.195, *P* = 0.031), hypertension (*χ*^2^ = 5.301, *P* = 0.024), rupture (*χ*^2^ = 4.514, *P* = 0.047), and postoperative radiological infarction (*χ*^2^ = 12.611, *P* = 0.001) were significantly related. The binary logistic regression analysis revealed that postoperative radiological infarction (odds ratio (OR) = 6.033, 95% confidence interval (CI) (1.057–34.437), *P* = 0.043), rupture (OR = 57.966, 95% CI (1.112–3020.66), *P* = 0.044), and hypertension (OR = 24.462, 95% CI (1.385–431.917), *P* = 0.029) are the potential independent predictive factors (Table 3).

**Table 3:** Univariate and multivariate analysis for postoperative RI and outcome of fPCA aneurysm

Dependent variable	Independent variable	Univariate		Multivariate	
		*P* value	*t*/*χ*^2^	*P* value	Odds ratio (95% CI)
**Radiological infarct**	Aneurysm size	0.015*	− 2.482	0.214	1.096 (0.949–1.265)
	fPCA type	0.027	5.873	0.06	0.264 (0.066–1.056)
	Intraoperative temporary clipping	0.043^&^	4.794	0.193	0.343 (0.068–1.719)
**3-month outcome**	Aneurysm size	0.016*	− 2.492	0.056	1.235 (0.995–1.532)
	Age	0.031*	− 2.195	0.128	1.104 (0.972–1.255)
	Hypertension	0.024^&^	5.301	0.029	24.462 (1.385–431.917)
	Rupture	0.047^&^	4.514	0.044	57.966 (1.112–3020.66)
	Postoperative radiological infarction	0.001^&^	12.611	0.043	6.033 (1.057–34.437)

*t test

^&^Fisher’s exact test

## Discussion

Morphology and hemodynamics have been revealed to play important roles in the generation and rupture of intracranial aneurysms [14]. As reported, the fPCA is a common variant of Willis circulation in the whole population [1, 2, 15, 16]. Generally speaking, fPCA contributes to a particular architecture of Willis circle and is more likely to gestate aneurysms due to disturbed hemodynamics distribution [17, 18]. In a comparative analysis about PComA, Thiarawat et al. revealed that ipsilateral fPCAs get a higher possibility to harbor aneurysms than other hemodynamics types (42% vs 14%, *P* < 0.001) [19]. Similarly, in this series, we found the fPCA aneurysm patients accounted for 19.1% (74 out of 388) of the population with PComA aneurysms.

The ipsilateral fPCA feeds the whole territory, which is formerly supplied by conventional posterior cerebral artery, and exposes to more violent impact of blood flow. In this case, aneurysms grow more easily. In case of blocking or injury of fPCA, infarction develops and patients tend to suffer from poor outcomes because of the lack of compensatory from posterior cerebral circulation [20]. Cerebral infarction is strongly correlated with angiographic vasospasm for ruptured aneurysms. The constituent and accompanying inflammatory could cause a strong constriction of the blood vessels. But in some cases, angiographic vasospasm was not detected. It is because that there were still other reasons, such as small-vessel spasm and micro-thromboembolism, cortical spreading ischemia, intraoperative hypotension, coagulopathy, perforator occlusion attributable to the aneurysm-securing procedure, or a complication of catheter angiography. Therefore, in order to prevent the occurrence of postoperative cerebral infarction, blood pressure should be controlled steadily throughout the perioperative period to ensure effective cerebral perfusion pressure, careful handling should be recommended to protect perforating blood vessels during operation, and hemostatic agents should be used cautiously.

In this study, we described clinical data of 74 aneurysms in the origin site of posterior cerebral artery firstly (Table 1). We noticed some common features as aneurysms of other types, such as a female preponderance (56 vs 18) [21, 22], high prevalence of hypertension (62.2%) [23], and high rupture rate, which are in accordance with the previous reports.

The architecture of fPCA is easier to cause hemodynamic aberrance. Due to the autocephalous architecture, injury or occlusion of the fPCA may bring about severe occipital infarcts and subsequent clinical complications, such as homonymous hemianopsia, alexia, aphasia, and hemiachromatopsia [16]. As suggested by the previous study, endovascular occlusion of an aneurysm and its parent artery is of high risk for patients with fetal-type PCA aneurysms [24]. In this surgical series of 74 cases, 19 (25.7%) patients presented postoperative radiological infarction. Of them, 14 are from the *perforator* PComA, 2 have distal PCA infarct, and 3 share both. There are 4 that refer to MCA or ACA territory infarcts in this series. The univariate analysis (*χ*^2^ = 5.873, *P* = 0.027) and binary logistic regression (OR = 0.264, 95% CI (0.066–1.056), *P* = 0.060) demonstrated that full type of fPCA tend to be a predictive factor for postoperative radiological infarction.

Endovascular embolization of the PComA aneurysms has been widely accepted as an effective treatment modality. But endovascular treatment of fetal PComA faces some challenges, specifically for the ruptured ones. Roy et al. reported an independent association between incomplete occlusion and fPCA configuration [25]. Wallace et al. reported a complication rate of 14% and overall complete occlusion rate of 33% [26]. In this series, all aneurysms were completely clipped and 11 (15.0%) suffered complications.

The follow-up data at 3 months after surgery showed that 63 patients (85.1%) got favorable prognosis with the GOS of 4 and 5. The regression analysis showed that postoperative radiological infarction, rupture, and hypertension tend to be the independent predictive factor for poor prognosis at 3 months after discharge.

Our study has some limitations. First, this study is a retrospective study and the number of patients is small, thus leading to bias. Prospective and a larger number of patients are needed to confirm the results. Second, the patients are screened in one institution and selection bias may have affected the veracity of outcome.

## Conclusion

In conclusion, we report a series of patients harboring aneurysms originating from the fPCA. Surgical clipping is a reliable strategy. Patients with full fPCA type of PComAs got preference to develop postoperative radiological infarction. And postoperative radiological infarction, rupture, and hypertension history predicted poor prognosis at 3 months after surgeries.

## Data Availability

Please contact author for data requests.

## Abbreviations

fPCA: Fetal posterior cerebral artery
PComA: Posterior communicating artery
ICA: Internal carotid artery
CT: Computerized tomography
CTA: Computerized tomography angiography
CTP: CT perfusion imaging
DSA: Digital subtraction angiography
OR: Odds ratio
CI: Confidence interval
